# The Blood: Its Progress and Function

**Published:** 1867-09

**Authors:** Rufus King Browne


### ARTICLE VI.

### *The Blood: Its Progress and Function.*

By Professor Rufus King Browne, M.D.

The Mammalian blood, consists of a very viscous liquor, and two sets of soft-solid bodies—the white and red corpuscles. Of the anatomical character of the former, I have already spoken. I shall now briefly consider the results of observation, upon the different rates of progress through the blood channels, of these two constituents. The current of the blood passes through a set of endless or closed pneumatic tunnels, of fleshy, contractile and resilient substance, filled not quite to repletion, the arteries and their extensions, the capillaries and veins. One of the earliest facts which forces itself upon the notice of the careful and appreciative observer of the circulation is, that there is the most notable difference in the rate and passage of the liquid of the blood, and its soft, solid constituents—or corpuscles, especially the red ones. The red-corpuscles do not pursue their way through the column of fluid, at the rate of the latter, but with a rapid motion or forward movement within it. The plasma moves at a far slower rate. The so-called "still layer" observed in microscopic examination of the current of the blood, within the capillaries of a living animal, is not at all what the designation describes, but is the slow moving plasma. The red-corpuscles dart through the plasma each in its own track, until they reach the capillary flow, where they move at a still different rate from the there more slowly moving plasma. There has always been with physiologists an ill-defined notion, that the red-globules were "destroyed" in the organism;—a supposition which has existed in the form of the notion that they are disintegrated or broken up in some particular organ of the body. But this supposition, on the other hand, has been but little respected by other physiologists, who have as an alternative, maintained that these were as permanent elements, as any of the anatomical constituents of the body, organized in tissue. The first notion is undoubtedly erroneous, for it is apparent to the least reflection, (which seemingly those who maintain it have never given to the subject) that a destruction or disintegration, bodily into fragments, of these bodies, would constitute a permanent disability of the destroying organs, to dispose of the soft solid debris of such a process. But, this objection does not exist against a gradual and progressive, continued, process of *dissolution*, accomplished not in virtue of the destroying function of any organ, but in virtue of the remittent partial oxidation and *de*oxidation of these bodies, during each transit through the circuit.

During their course, through the plasma, the red blood-globules continually yield to it a part of the oxygen they take up in the pulmonary circuit—while the plasma itself yields to the tissues, a part of its highly oxidized substance to repair, by molecular action, the substances constituting the waste. Although we just spoke of the highly *oxidized* plasma, yet the form in which this yielding of the oxygen of the globules to it, is performed, is not an *oxidising* one—*not* the formation of a compound with *oxygen*, but one in which the oxygen is in association with the plasma, and this is undoubtedly the state, namely, of association, in which it is yielded to the tissues.

The well known truths of chemistry have long and generally, if not always, been misapplied in the notion that, the impletion of oxygen is a building or repairing process, for it is exactly the converse of this, being a process of preparing *waste* material. It is for this process that the plasma containing oxygen is transuded to the tissues. It is a fact well known, that in the ultimate or cellular portion of the lung tissue, amid which everywhere the capillaries of that structure run, the blood channels hold a far larger proportion of red-globules, than elsewhere. This is not to be accounted for on the supposition, that any *portion cf* the plasma has been *retarded*, but only, by recognizing the fact, that the globules have made the round of the circuit, with greater celerity, than the corresponding plasma, which received its impulsion from the left ventricles.

This phenomenon is consequent on the fact, that the blood-tunnels are never filled to repletion, or to the extent of their full capacity, by the volume of plasma, so that as has been often noticed, there occur very wide differences in their comparative fulness, in different parts of the system. In one capillary district, at a given time therefore there may not be more than half, or less, the actual quantity, that there is in another, or others, of precisely equal holding capacity or calibre.

This remarkable difference of quantity in that portion, of the arterial system, which (with its walls) constitutes the heart, is regular and intermittent; but it also exists, at all times, in various parts of the vascular system, as compared at one moment with another.

The flow of blood through the lungs is not constant but remittent. With each diminution or "collapse" of the size of the lungs, a very remarkable and hitherto entirely unnoticed change takes place, in its vascular or capillary portions. During each enlarged or inspiratory state of the lungs, the blood-hollows, are at their normal capacity and condition for the free passage of the blood ; but during expiration, the capillaries return to a state of comparative compression, by contorting upon themselves,—a phenomenon not easy to demonstrate and this unfortunately it seems as yet not practicable to obtain, for the *action* of the lung is one which cannot be observed with the microscope.

So far, as I know no observer has yet hinted or even imagined the fact, that the capillary districts of the lungs, must undergo very remarkable mechanical and anatomical *changes* in their adjustment, as an arrangement of structure, in the periods of the acts of *in*spiration and *ex*piration.

The lungs are continually passing, without intermission, into either one of two widely and unlike anatomical characters as organs : *from* one of comparatively small size and air-holding calibre, *to* another of comparatively great size or calibre. Between these two perfectly reciprocal characters of form, size or dimensions, there is no state of cessation, rest, or stasis, but an undivided continuity of movement, to the least, or the greatest of their dimensions. Between the end of *in*spiration and beginning of *ex*piration there is no cessation. They are in a constantly dynamic condition as compared with a *static* one.

This change in their dimension, size, contour and calibre, is a change in the arrangement of all the tissues comprising these structures. The fact needs but once to have been conceived and stated, to secure instant assent,—to be admitted as a fact. But to demonstrate the exact character, mechanically and anatomically considered, of this change, is the difficulty I have alluded to, as one we are not yet able to overcome. Of the fact, no faintest recognition, has yet been made, for the various designations uniformly in use, intended as descriptive of this constant progress of passing from the greatest to the least size, from one anatomical character and shape to a different one, represent a meaning quite antagonistic. The terms "compression" and "dilation," "collapse" and "expansion," show a notion, entirely at variance with the above truth. A demonstration of the fact by the microscope would demand a complete suspension of *the act* of anatomical change, and this seems impossible.

But in the absence of this inspection, we can readily conceive the extent of this alteration of shape, since it must take place, in every direction, represented by lines, radiating towards both the pleur side and the air-containing or epithelial side, of the entire cones of tissue. It consists of rapidly progressive swelling out *in every direction* of the elements of tissue, passing into an equally rapid progressive return to the minimum size. This movement cannot be at all similar, except in the very gross cubic measure sense, to a change from occupying more, to filling *less* space—to a movement of "distension" as in the case of a membranous sac or vesicle, the uniform wall of which recedes outward, as the entering substance progressively occupies its hollow interior. But on the contrary, it is a movement of the elements of tissues toward the air filled spaces, as well as in other directions.

Now while we are as yet unable, to *see* this act, and thus represent it by an image of the advancing stage, and alteration of the form of the tissues, yet we can, having once mastered the idea, conceive the probable steps, and thus assure ourselves with entire certainty that some equivalent to these steps, *must* occur.

Everywhere throughout the general capillary system, the calibre of the capillaries, is very much greater than the actual size of the current of fluid they contain, except indeed in local morbid states.

The inner surfaces of their exceedingly attenuate walls are easily brought into contact or apposition, by the slightest pressure upon their outer surfaces. This bringing into contact of the inner surfaces, may easily cause an interruption of longer or shorter duration of the flow of their contents or of a portion thereof. But elsewhere in the capillary system no attenuation of form takes place, such as we are describing as probable in the capillary part of the lung structure. In the progressive extension, elongation or whatever other change of shape takes place, in the progressive enlargement and diminution of the whole tissue of the lungs, the capillaries must undergo the most change.

During the progress of the diminishing and diminished size, the capillaries may be irregularly curved or turned upon themselves. And if this be a correct statement of their course and shape in that state, in the enlarged or swelling stage, they must pass into a changed form, perhaps into a straightened and more direct course. These changes of form cannot occur without involving some effect on the flow of the fluid which occupies their hollows. These effects will vary directly as the anatomical character or shape of the vessels varies; but the minimum and maximum of them, will occur at the moment of transition, when the inspiratory state is being converted into the expiratory, and conversely. Corresponding to this change of anatomical character, the tide of blood, may be *stayed*, to be instantly succeeded by a free pushing or impulsion setting forward at an accelerated rate through the capillaries toward the heart. This followed by continuous accessions in unbroken consecutiveness by these succeeding anatomical changes, are of precisely such a character, as to cause consecutive fulness and depletion, or an approximation to either, the displacement from the plasma of carbonic acid, watery, and animal vapor,—the products of expiration,—on the one hand, and of the displacement from the air inwardly of the oxygen, on the other, find the proper anatomical conditions.

Brief and imperfect as this outline of the probable facts is, it is yet sufficient to force upon our apprehension, a sense of the great and hitherto wholly unsuspected change in anatomical character, which must with the constancy of the utmost regularity, take place in the consecutive, transition from one state of the lungs to the other. And a full understanding of the case, will undoubtedly afford a complete explanation of the exact physiological events upon which are based the absorption of the oxygen, and the partial removal from the blood of carbonic acid, and of the expiratory waste substance, emitted in the outgoing breath.

It is obvious, that the uniformly accepted estimation of the absorption of oxygen, by the blood, namely, that it passes atom by atom, in a continuous stream, into the blood, through the double partition between the air and the blood, formed by the walls (side to side) of the capillaries and the epithelium of the air cells, is not satisfactory. And of the several classes of agencies in operation to effect its entrance, perhaps none bear so important a part as the change in anatomical characters, I have adduced.

Unremitting attention to the known facts of the case, and constant endeavor to ascertain them in their entirety, have forced upon me a sense of the necessity of investigating the changes of anatomical character which, from moment to moment, the lung tissue is perpetually undergoing, as a condition of understanding the exact nature of the functions of respiration.

Whoever reflects upon this subject cannot fail to find himself concurring with my convictions. It is no valid form of dissent therefrom, to cite the want of actual demonstration. If the view I present fails, as a demonstration, it is to be admitted that it only fails as a *visual* demonstration, and not in a sufficiency of facts. The visual demonstration, it is apparent, is in itself seemingly hardly within the limits of possibility, but it may yet confirm the view I present.

### CORRESPONDENCE.

